# The Rotterdam Scan Study: design update 2016 and main findings

**DOI:** 10.1007/s10654-015-0105-7

**Published:** 2015-12-09

**Authors:** M. Arfan Ikram, Aad van der Lugt, Wiro J. Niessen, Peter J. Koudstaal, Gabriel P. Krestin, Albert Hofman, Daniel Bos, Meike W. Vernooij

**Affiliations:** Department of Epidemiology, Erasmus MC University Medical Center, P.O. Box 2040, 3000 CA Rotterdam, The Netherlands; Department of Radiology, Erasmus MC University Medical Center, Rotterdam, The Netherlands; Department of Neurology, Erasmus MC University Medical Center, Rotterdam, The Netherlands; Biomedical Imaging Group Rotterdam, Erasmus MC University Medical Center, Rotterdam, The Netherlands; Faculty of Applied Sciences, Delft University of Technology, Delft, The Netherlands

**Keywords:** Epidemiology, Population-based, Risk factors, Neuroimaging, Cohort study, Dementia, Stroke, Alzheimer’s disease, Microbleeds, White matter lesions, Infarcts, Cerebral blood flow, Diffusion tensor imaging, Genetics

## Abstract

Imaging plays an essential role in research on neurological diseases in the elderly. The Rotterdam Scan Study was initiated as part of the ongoing Rotterdam Study with the aim to elucidate the causes of neurological disease by performing imaging of the brain in a prospective population-based setting. Initially, in 1995 and 1999, random subsamples of participants from the Rotterdam Study underwent neuroimaging, whereas from 2005 onwards MRI has been implemented into the core protocol of the Rotterdam Study. In this paper, we discuss the background and rationale of the Rotterdam Scan Study. Moreover, we describe the imaging protocol, image post-processing techniques, and the main findings to date. Finally, we provide recommendations for future research, which will also be topics of investigation in the Rotterdam Scan Study.

## Introduction

Neurologic diseases in the elderly, such as dementia and stroke, will pose an ever increasing burden on societies over the next couple of decades [1–4]. Yet, effective therapeutic or preventive strategies are still lacking. In order to develop such strategies, knowledge on the etiology of these diseases is crucial. An important feature of neurodegenerative diseases is that structural and functional brain changes may be already present years before clinical onset and can be visualized using magnetic resonance imaging (MRI) [5–10].

Realizing this potential benefit, already in the 1990s, neuroimaging was implemented in several population-based studies to study the preclinical brain changes that ultimately lead to or may indicate an increased risk of developing clinically manifest diseases, such as dementia and stroke [7, 11–19]. However, in most of these studies neuroimaging was only performed in a subset of the population, resulting in limited sample sizes. More importantly, during the last two decades MR imaging has undergone huge improvements in hardware and software leading to higher field strengths, higher resolution, shorter scanning times, and more sensitive sequences. In addition, digital image analysis techniques have led to a new field of research aimed at automating and increasing through-put of image processing for better visualization and quantification of imaging findings. Taken together, these developments now allow for performing neuroimaging in larger sample sizes and using state-of-the-art imaging and processing techniques. In turn, this has paved the way for more in depth and thorough investigation of (more subtle) brain changes that can lead to neurological diseases.

It was in this light, that in 1995 the Rotterdam Scan Study was initiated to investigate risk factors and risk indicators of neurological diseases in the elderly using MR imaging to visualize the underlying brain changes and brain pathology. In 1995 and 1999, random subsamples of Rotterdam Study participants underwent neuroimaging in clinical scanners. From 2005 onwards, the Rotterdam Scan Study has been embedded within the core protocol of the Rotterdam Study [20], and a dedicated research scanner was installed in the Rotterdam Study research center.

In the current paper, we provide a general outline of the study population, scanning protocol, image post-processing and a discussion of the main findings of the Rotterdam Scan Study, with the main focus on the period from 2005 to 2015.

## Design and study population

The source population of the Rotterdam Scan Study originates from the Rotterdam Study [21], a population-based study in the Netherlands that aims to investigate causes and determinants of chronic diseases in the elderly. The Rotterdam Study (RS I) was initiated in 1990 with 7983 participants aged 55 years and over, who were interviewed and underwent physical exam at baseline and during follow-up visits every 3–4 years. In 2000, the cohort was extended with 3011 persons (RS II), who were aged 55 and over at that time. In 2006 the cohort was further extended with 3932 persons aged 45 years and over (RS III). The whole cohort undergoes re-examinations every 3–4 years. The total Rotterdam Study population encompasses 14,926 persons.

**Fig. 1:**
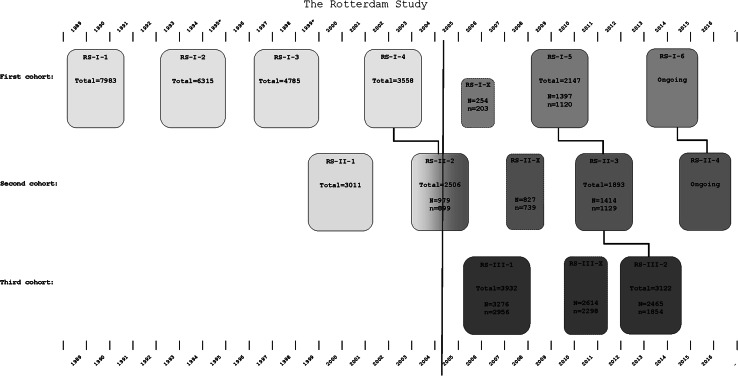
Overview of the sub-cohorts and examination visits of the Rotterdam Study, and imaging visits of the Rotterdam Scan Study. *Boxes* indicate examination visits of the three cohorts of the Rotterdam Study. *Boxes* with *solid* *colors* indicate visits, during which MRI imaging was conducted as part of the core protocol. Examination visits indicated with an ‘X’ indicate extra visits during which only MRI was performed. The red vertical line indicates the implementation of MRI on site in the core protocol of the Rotterdam Study. In 1995 and 1999 (indicated with *) 567 persons underwent MRI as part of the Rotterdam Scan Study outside the core protocol of the Rotterdam Study. ‘Total’ indicates the total number of persons taking part in that Rotterdam Study examination visit. ‘N’ indicates the number of persons that were eligible (no MRI contra-indications and no claustrophobia) and invited to take part in the Rotterdam Scan Study. ‘n’ indicates the number of persons that underwent brain MRI in the Rotterdam Scan Study

Figure 1 shows an overview of the various Rotterdam Study cohorts, the time of their (re-)examination visits, and the implementation of MRI-scanning in the core protocol of the Rotterdam Study in 2005. Initially, we invited random persons from the second visit of RS II to undergo MRI. Subsequently, we have scanned all eligible and consenting participants from the first visit of RS III and fifth visit of RS I. Currently, persons from the fourth visit of RS II are undergoing scanning. Of all persons taking part in the Rotterdam Study, those with MRI contra-indications are considered not eligible for the Rotterdam Scan Study. Furthermore, persons suffering from claustrophobia are also not included. Because the throughput of performing MR imaging has been higher than that of the Rotterdam Study (56 MRI slots per week versus 36 slots for regular study center visits), we were able to invite additional subsets for re-scanning. As such, we re-invited participants from RS I in 2006, RS II in 2008, and RS III in 2010 outside their regular visits for the Rotterdam Study. As a result, some of the participants are already undergoing their fourth MRI-exam.

Therefore, as of July 2015, a total of 12,174 brain MR-scans have been obtained on the research scanner in over 5800 individuals.

## Scan protocol

In 2005, a 1.5T MRI unit (General Electric Healthcare, Milwaukee, USA, software version 11x), dedicated to the Rotterdam Scan Study, was installed in the Rotterdam Study research center. Besides the possibility of high throughput image acquisition, this enabled us to leave acquisition parameters unchanged by excluding software or hardware upgrades in order to ensure data stability and comparability over time. The MRI unit was fitted with a dedicated 8-channel head coil (best coil configuration available at time of installation) and the possibility for parallel imaging using the array spatial sensitivity encoding technique (ASSET).

Maximum total examination time (from arrival of one participant in the MRI suite until the next) was initially set at 45 min, in order to accommodate the MRI acquisition into the generic workflow of the Rotterdam Study. Later, this became 50 min due to the addition of a resting-state functional MRI sequence (rs-fMRI), which is discussed in detail below.

In the current scan-protocol we carefully balanced the restrictions of time, costs and inconvenience for the participants with the relevance and quality of the acquired imaging data. To ensure participant compliance and reproducible image quality (reduce motion artefacts) an acquisition limit of 6 min per sequence was chosen.

To facilitate easier applicability of the current MRI protocol by radiology technicians, we chose to use the standard brain imaging package delivered by the system manufacturer instead of custom developed sequences.

The MRI sequences were chosen based on the primary variables of interest, i.e.:

1. quantitative measures of brain tissue volumes and volumes of various neurostructures (e.g. hippocampus)
2. quantitative assessment of white matter lesions (WMLs),
3. qualitative assessment of brain infarcts (lacunar and cortical) and microbleeds,
4. quantitative assessment of white matter microstructural integrity and connectivity,
5. quantitative assessment of total cerebral blood flow,
6. quantitative assessment of functional brain networks.

In designing the protocol, we tried to meet both the time constraint and the contrast and resolution requirements. When possible, we preferred 3D over 2D sequences because of higher signal-to-noise ratio (SNR), which enables the acquisition of smaller voxel sizes. Yet, acquisition time, sensitivity to motion and blurring artefacts did not allow 3D acquisition in all sequences. For each sequence, we adjusted the imaging parameters during optimization procedures to obtain a specific target resolution with adequate SNR (≥25) for tissues in the center of the brain while scan time was limited to 6 min for each sequence.

Since mid-2011, the structural MRI protocol has been extended with a resting-state functional MRI (rs-fMRI) sequence, further described below. Despite the original aim to limit scan time to 6 min per sequence, this functional scan requires 8 min to obtain adequate resting-state data.

The resulting protocol is presented in Table 1 with all the relevant imaging parameters and the execution order listed. The protocol starts with a three-plane localizer, executed with the shimming option enabled. For subsequent sequences shimming is turned off to accelerate receiver adjustments. Morphological imaging is performed with T1-weighted (T1w), proton density-weighted (PDw) and fluid-attenuated inversion recovery (FLAIR) sequences. The combination of different MR contrasts provided by these sequences can be used for automated segmentation of brain tissue and WMLs (see section on processing). For the purpose of segmentation, the T1w scan is acquired in 3D at high in-plane resolution and with thin slices (voxel size < 1 mm3). A 3D T2*-weighted gradient-recalled echo (GRE) scan is used to image cerebral microbleeds. For this sequence a TE > 30 ms was selected to obtain stronger T2*-weighting. For registration purposes, the same slice thickness with a lower in-plane resolution as compared to the 3D T1w scan is used. Parallel imaging is applied for this sequence to stay within the 6 min scan time limit.

**Table 1:** The magnetic resonance imaging protocol used in the Rotterdam Scan Study

Sequence	Comment	Mode	Readout module	Time (min:sec)	TR/TE (ms)	TI (ms)	BW (kHz)	Flip angle (degrees)	Number of slices	Slice thickness (mm)	FOV (cm^2^)	Matrix
Scout (1)	Positioning	2D	GRE	0:07	7.9/1.8		31.25	30	3	4	30	256 × 256
Scout (2)	Localizer for 2D phase contrast scan; VENC = 60 cm/s	2D	GRE	0:12	24/9.0		8.06	10	1	60	32	256 × 160
PDw		2D	FSE	6:09	12,300/17.3		17.86	90–180	90	1.6	25	416 × 256
2D Phase Contrast	Carotid and basilar flow; VENC = 120 cm/s, NEX = 8	2D	GRE	0:51	20/4.0		22.73	8	1	5	19	256 × 160
rs-fMRI		4D	EPI	7:44	2900/60		7.81	90	31	3.3	21	64 × 64
T1w		3D	GRE	6:24	13.8/2.8	400	12.5	20	96 (192)	1.6 (0.8)	25	416 × 256
FLAIR		2D	FSE	6:25	8000/120	2000	31.25	90–180	64	2.5	25	320 × 224
ASSET	Coil sensitivity correction data for calibration of parallel imaging	2D	GRE	0:06	150/1.8		31.25	70	39	10	30	32 × 32
DTI	25 directions; b = 1000 mm^2^/s, b_0_ NEX = 3	2D	EPI	3:44	8000/74.6		250	90–180	39	3.5	21	64 × 96
T2*w		3D	GRE	5:55	45/31		14.71	13	96 (192)	1.6 (0.8)	25	320 × 224

*PDw* proton-density weighted, *T1w* T1-weighted, *FLAIR* fluid-attenuated inversion recovery, *ASSET* array spatial sensitivity encoding technique, *DTI* diffusion tensor imaging, T2*w T2*-weighted, *rs-fMRI* resting state functional MRI, *GRE* gradient-recalled echo, *FSE* fast spin echo, *TR* repetition time, *TE* echo time, *TI* inversion time, *BW* bandwidth, *FOV* field of view; *VENC* velocity encoding, *NEX* number of excitations

Diffusion tensor imaging (DTI) is used to quantitatively assess white matter microstructural integrity [22, 23]. For this 2D DTI scan, we use an echo planar imaging (EPI) readout with gradients (b = 1000 s/mm^2^) applied in 25 directions [22, 23]. The b = 0 s/mm^2^ image is collected with NEX = 3. The number of gradient directions, i.e. 25, was chosen to best fit the optimized protocol by Jones et al. [22, 23] whilst remaining within time limits and maximum number of slices permitted by the scanner. To minimize geometrical distortions, the number of frequency encoding points was set to 64 and parallel imaging was applied with an acceleration factor of 2, with an imaging matrix of 64x96, providing a voxel size of 3.3 × 2.2 × 3.5 mm^3^.

An ungated 2D GRE phase contrast flow measurement is applied for assessment of total cerebral blood flow [24], which has shown to be fast and accurate [24]. A 2D thick slab projection phase contrast angiographic localizer (60 mm thick, velocity encoding (VENC) = 60 cm/sec) is positioned sagittally to determine the location of the carotid and basilar arteries. Next a thin slice perpendicular to all three vessels at the level of the precavernous internal carotid artery is positioned (VENC = 120 cm/s, slice thickness 5 mm, NEX = 8). Flow velocity data can be calculated from the phase difference images as described before [24]. For rs-fMRI, subjects are instructed to lie still with their eyes open, and not to fall asleep. T2*-weighted echo planar images (EPIs) are acquired with 3.3 mm isotropic voxels, and a total of one-hundred sixty volumes.

**Fig. 2:**
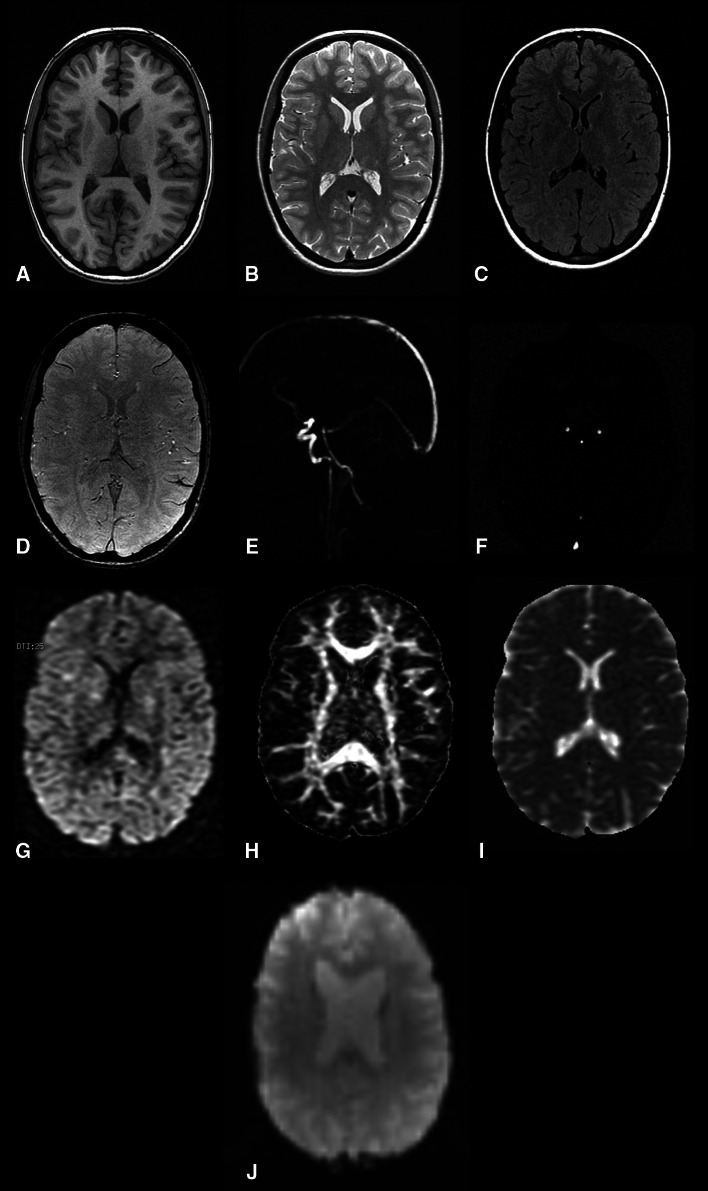
Depiction of the images acquired using the MRI protocol. First row: T1-weighted (*a*), proton-density-weighted (*b*), and fluid attenuated inversion recovery (*c*) images. Second row: T2*-weighted (*d*) image, sagittal scout for the 2D phase contrast measurement (*e*), and the resulting flow image (*f*). Third row: example of an image acquired using diffusion weighted imaging (*g*), map of fractional anisotropy (*h*), map of mean diffusivity (*i*), and resting-state functional MRI (*j*)

Figure 2 illustrates an example of the different sequences acquired in the final protocol. The sequence acquisition order was chosen in a way to provide adequate reconstruction speeds and to eliminate delays. The 3D T2*w GRE scan was the last sequence executed in the protocol pipeline because of the long reconstruction time necessary for parallel imaging and the resulting lag time in scan execution.

For quality check, a daily quality assessment (measuring transmit gain, center frequency and SNR) is performed by technicians using a phantom. Additionally, weekly measurements of echo-planar stability, isocenter reliability and accuracy of absolute scaling along the cardinal axes (x,y,z) are performed. Regular scanner maintenance is performed by the manufacturer and results are filed in a log.

Furthermore, interscan reproducibility measurements have been performed by re-inviting study participants (n = 20–30) within on average 2 weeks after initial examination for repeat MRI.

## Image processing

Within the context of the Rotterdam Scan Study, a standardized image analysis workflow is being developed, validated and applied to all imaging data, to enable the objective, accurate, and reproducible extraction of relevant parameters describing brain anatomy, possible brain pathologies, and structural and functional brain connectivity from multispectral MRI data. In the following paragraphs, we briefly describe the different quantitative image analysis methods that have been developed and/or employed within the Rotterdam Scan Study.

### Image pre-processing

Prior to analysis, a number of pre-processing steps are performed. For multispectral image analysis, the different scans are spatially registered using rigid registration. Subsequently, the brain is extracted from the scan. Hereto a manually segmented brain mask—which excludes among other things, the cerebellum, the eyes, and the skull—is non-rigidly registered to the T1-weighted image using Elastix [25].

Finally, scans are corrected for intensity non-uniformity using the N3 method [26]; non-uniformity correction is carried out within the brain mask.

### Brain tissue segmentation

**Fig. 3:**
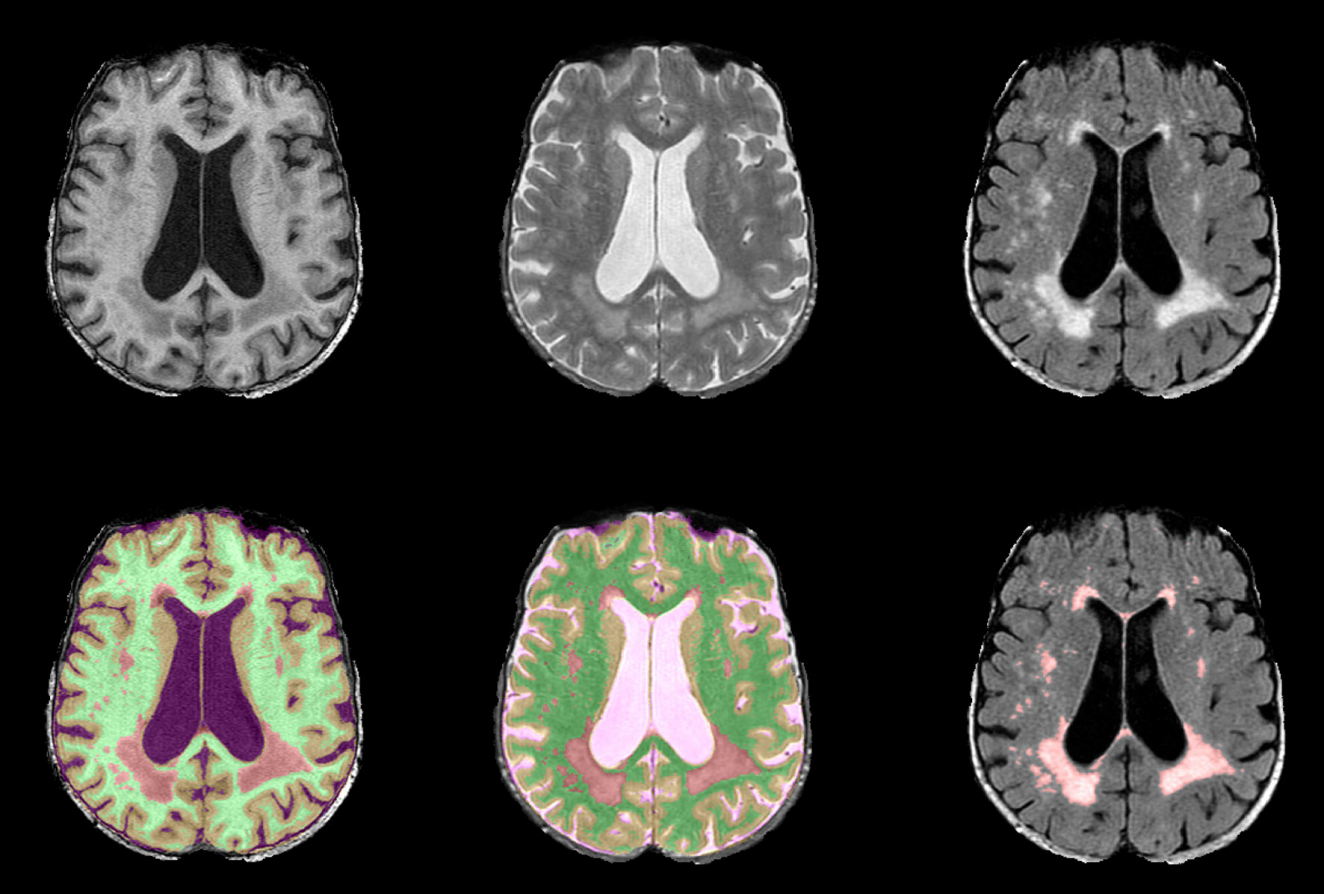
Example of brain tissue segmentation. *Left column* T1weighted sequence with k-nearest neighbor based tissue segmentation into grey matter (*orange*), white matter (*green*), cerebrospinal fluid (*purple*), and white matter lesions (*red*). Middle column: Proton-density weighted sequence with similar tissue segmentation. *Right column* FLAIR-sequence with white matter lesion segmentation (*pink*)

Automated brain tissue segmentation on MRI has received considerable attention [11, 27–32]. An important distinction that can be made is whether methods are supervised (i.e. they depend on annotated training data), or unsupervised. In the Rotterdam Scan Study, we use a supervised approach, based on k-nearest neighbour (kNN) segmentation. In kNN segmentation, image voxels are assigned labels (grey matter (GM), white matter (WM), cerebrospinal fluid (CSF) or background (BG)) based on the most similar voxels in the training data. Similarity here depends on the distance in normalized MR image intensities. We have both investigated segmentation based on T1w images, and multispectral MRI data (T1w and PDw images). Manual segmentations by two observers of six T1w datasets (the PDw dataset is implicitly segmented after rigid registration to the T1w datasets), that include labels for GM, WM, CSF, and BG, were used as training data [27, 33]. This brain tissue segmentation method has been extensively evaluated within the context of the Rotterdam Scan Study, showing good accuracy and reproducibility [33, 34]. An example of the automated tissue segmentation is shown in Fig. 3.

To facilitate more regionalized analysis of total brain, WM and GM volumes, individual lobes have been segmented. This is achieved by non-rigidly registering a template image in which the lobes have been manually outlined [35–37]. Finally, T1-weighted MR images have been used to calculate subcortical structures and thickness of the cerebral cortex using a model-based automated procedure using Freesurfer image analysis suite (http://surfer.nmr.mgh.harvard.edu/) [38, 39].

### White matter lesion classification

The brain tissue segmentation methods described above have been complemented with WML segmentation [40]. Hereto, both the brain tissue segmentation, and the FLAIR image are used. In the brain tissue segmentation, possible WMLs are misclassified as GM with a ring of WM voxels. In the FLAIR image the WMLs are hyperintense. We therefore process the histogram from the FLAIR image intensities of all voxels that are classified as GM, to estimate the mean and standard deviation of true GM voxels. Subsequently, WML voxels are extracted by intensity thresholding, where the threshold depends on the estimated GM distribution. False positives are removed by excluding voxels which are not sufficiently connected to the white matter. The different parameters (intensity threshold, and quantitative definition of not being sufficiently connected) have been optimized on a large reference dataset. The method has been quantitatively evaluated [40] and has successfully been applied to the whole cohort [41]. Visual inspection of the results indicates that the method is robust, with approximately only 4 % of the scans containing false positive or false negative WMLs. Figure 3 demonstrates the automated WML segmentation result.

### Brain structure segmentation and shape analysis

Within the Rotterdam Scan Study, we have developed a graph cut framework for neurostructure segmentation [42] combining atlas registration and statistical models of image appearance [43], which currently has been implemented for hippocampus segmentation. The developed framework utilizes twenty manually outlined hippocampi (atlases) [44], which are used both for atlas registration, and for training the statistical image appearance models. The twenty atlases are non-rigidly registered to an image to be segmented, after which by averaging a spatial probability map is obtained which indicates the likeliness of a voxel to belong to the hippocampus. Within a graph cut framework, this information is complemented by the likeliness that a voxel is part of the hippocampus based on intensity information, for obtaining a segmentation. The method has been shown to improve on existing manual hippocampus segmentation techniques [43], and has been applied to a number of studies [45, 46]. Recently, we have extended the hippocampus method to also include more informative appearance models [47]. The graph cut framework developed for hippocampus segmentation has additionally been used for ventricle segmentation [48], and segmentation of the cerebellum [49, 50].

Based on the hippocampus segmentation, we also have developed a method to quantify hippocampal shape, and demonstrated that the combination of hippocampal volume and hippocampal shape performed better on the prediction of dementia than when just using volume [51].

### Diffusion tensor imaging (DTI): global and tract-based analysis

DTI enables measurement of the microstructural integrity of white matter. Within the Rotterdam Scan Study, a number of image analysis techniques have been employed and developed for the analysis of DTI data. These include conventional global and regional analysis of DTI-derived measures such as Fractional Anisotropy (FA) and Mean Diffusivity (MD) [52], and tract-based analysis of FA and MD [53]. Global and regional analysis of FA and MD has been performed using the FSL toolbox [54], and consisted of Eddy current correction, head motion correction, skull stripping and tensor model fitting. As discussed below, DTI data were registered with the other imaging data to study relations between atrophy, WMLs, and DTI-derived measures.

Tract-based analysis of DTI enables a more localized comparison of FA and MD between groups. In the Rotterdam Scan Study, tract-based analysis has been achieved using tract-based spatial statistics (TBSS) [55] a technique that creates a common skeleton of the white matter tracts from a series of images, onto which for each individual the local maximum FA value is projected. This enables robust voxel-wise statistical analysis of the microstructural integrity of white matter across persons [53].

**Fig. 4:**
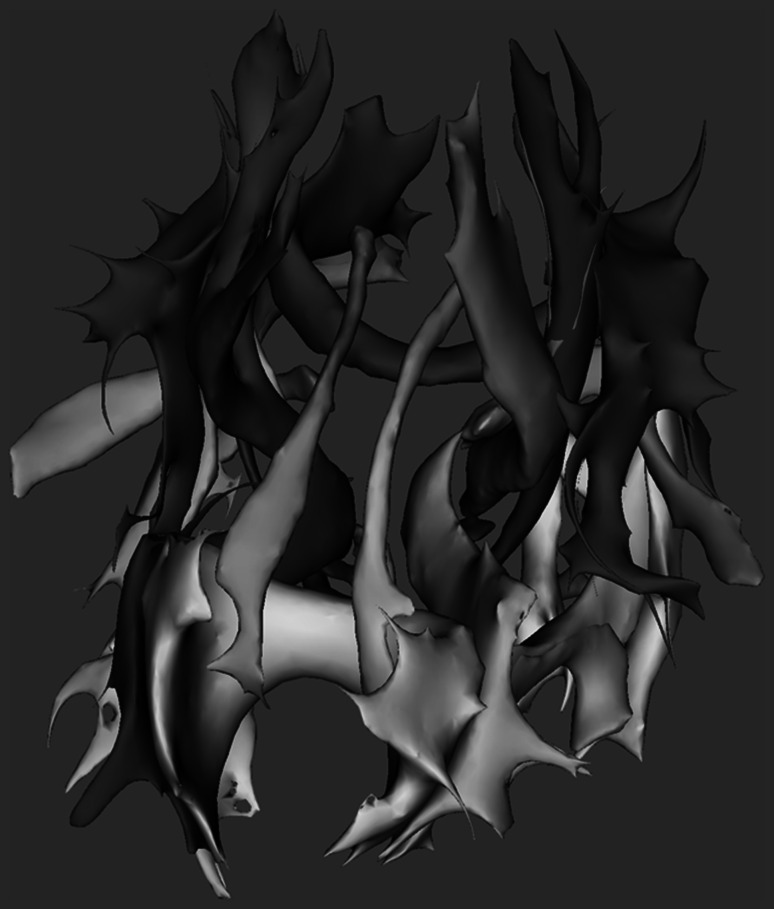
Example of the automatic analysis of diffusion MRI characteristics in 23 white matter structures

Since the projection step in TBSS may break topological consistency of the transformed images, we investigated whether the correspondence step in TBSS could be replaced by non-rigid registration. We evaluated performance of non-rigid registration to the conventional TBSS approach by performing tractography in native space and measuring the ability of the correspondence step in creating similarity in tractography results in 23 white matter structures in a common template space. It was shown that both non-rigid registration using Elastix [56] and FMRIB’s Nonlinear Image Registration Tool (FNIRT) [57] outperformed the conventional TBSS analysis [58]. Furthermore, the approach enables the automatic analysis of diffusion MRI characteristics in 23 white matter tracts. An example of 23 automatically generated white matter tracts on a subject of the Rotterdam Scan Study is shown in Fig. 4.

### Diffusion tensor imaging: connectivity analysis

Using deterministic or probabilistic tractography, DTI can also be used to study structural connectivity of the brain. In order to compare structural connectivity across persons in the Rotterdam Scan Study, we developed a novel framework, SAMSCo, which enables construction of weighted structural brain connectivity networks which can be effectively analyzed using statistical methods [59, 60]. The weighted networks are obtained using a minimum cost path (mcp) method with an anisotropic local cost function based on the locally estimated diffusion tensor weighted images. Start and end regions of the mcp were defined by a Freesurfer segmentation [61, 62] of subcortical structures and cortical parcellation. Using a re-scan on 30 persons, good reproducibility of the connectivity maps was shown [34].

### Resting-state functional MRI: resting-state networks

Preprocessing and analysis of rs-fMRI data is performed using the FMRIB software library (FSL, http://www.fmrib.ox.ac.uk/fsl/). Resting-state fMRI volumes are registered to the individual’s structural scan and standard space using FNIRT [57]. A single-subject independent component analysis (ICA) [63] approach is used to decompose the acquired rs-fMRI data into various components of resting-state activity in each participant. Low-frequency drifts and motion components are handled with MCFLIRT and temporal filtering [63, 64]. Next, we applied an automatic component classification using FMRIB’s ICA-based Xnoiseifier (FIX) for the discrimination between true signal versus noise components [65, 66]. Next, using dual regression [67], spatial maps of various resting-state networks, including the default mode network, are derived for each participant. These maps are then used to generate measures of functional connectivity and clusters of activation within each network, and later also to make voxel-based comparisons.

## Visual ratings

### Scan quality and incidental findings

Each MRI scan that is acquired is visually examined by a research physician in the Rotterdam Scan Study. During this visual inspection, the MRI scan is rated for quality and the presence and severity of motion artefacts or signal inhomogeneity (for example due to metallic implants) is recorded. Furthermore, each scan is evaluated by trained research physicians for presence of incidental findings, i.e. abnormalities of potential clinical relevance that were previously unknown, that are unexpected and that are unrelated to the purpose of the scan [68, 69]. All potential findings are recorded in a database and are in a later stage evaluated by an experienced neuroradiologist. Referral of participants for further medical examination occurs in accordance with an expert-defined protocol [68].

### Visual check of automated processing performance

**Fig. 5:**
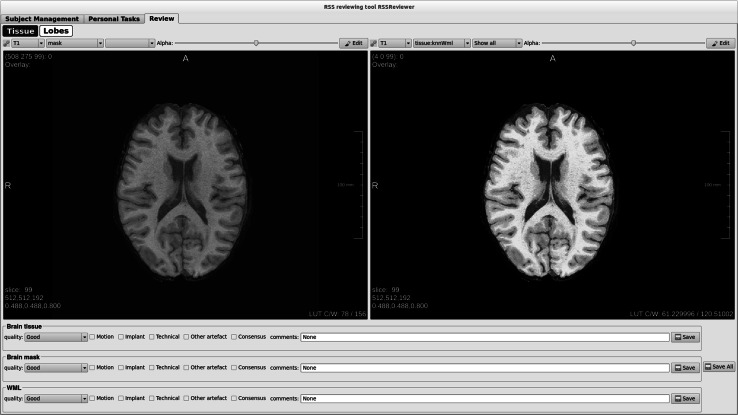
Screenshot of the tool which enables the visualization of the original scan with the image processing results. On the *left screen*, the mask for total intracranial volume is shown. On the *right screen* the rater can evaluate the tissue segmentation. In the *lower panel*, the rater can indicate the quality of the mask, the brain tissue segmentation and the white matter lesion segmentation

Though post-processing for tissue segmentation and structure segmentation takes places fully automated and without user interaction, all end results are visually checked for performance. For example, small motion artefacts in the FLAIR sequence that do not necessitate exclusion of an MRI scan may interfere with WML segmentation and cause false positive lesions after automated segmentation; or brain masking may result in minimal inclusion of dura or skull. For manual inspection, a dedicated tool has been developed in MevisLab^®^ enabling the visualization of the original scan with the image processing results (Fig. 5). Editing tools are available to adjust the segmentations if necessary. After visual inspection, manual editing of any errors is needed in less than 10 % of scans, depending on the type of post-processing. Furthermore, less than 1 % of scans are excluded based on artefacts that are only discovered after automated post-processing (for example motion or susceptibility artefacts in diffusion tensor images, which are not apparent in the raw unprocessed data).

### Cerebral small vessel disease

Infarcts are rated on FLAIR, proton density-weighted, and T1-weighted sequences. Lacunar infarcts are defined as focal lesions ≥3 mm and <15 mm in size with the same signal characteristics as CSF on all sequences, and (when located supratentorially) with a hyperintense rim on the FLAIR sequence [68]. Lesions ≥15 mm in size, but otherwise similar, are rated as subcortical infarcts. Infarcts showing involvement of cortical gray matter are classified as cortical infarcts. We further distinguish cortical infarcts into small and large infarcts based on their size [70].

All 3D T2* GRE scans are reviewed for the presence, number, and location of cerebral microbleeds. Microbleeds are defined as focal areas of very low signal intensity on T2*-weighted imaging that are not accompanied by evident signal abnormality on other structural sequences [71]. Microbleed location is categorized into one of three locations: lobar (cortical gray and subcortical or periventricular white matter), deep (deep gray matter: basal ganglia and thalamus, and the white matter of the corpus callosum, internal, external, and extreme capsule), and infratentorial (brainstem and cerebellum) [71]. Intraobserver and interobserver reliabilities for microbleed rating are very good (κ = 0.85–0.87 [72]) and review of the intial ratings by an experienced neuroradiologist yielded very high accordance as well [72].

### Virchow-Robin spaces

Virchow-Robin spaces (VRS), or enlarged perivascular spaces, are primarily rated on the PDw-sequence according to a standardized protocol [73]. In short, VRS are identified by their linear, ovoid, or round shape depending on the slice direction and are considered dilated when their diameter is ≥1 mm. VRS are assessed in 4 brain regions: the semioval center, the basal ganglia, the hippocampi, and the mesencephalon. Raters determine the amount of dilated VRS for each region, with a maximum of 20 per region. Because the semioval center and basal ganglia are visible on multiple slices, the rating is done on a single, predefined slice to decrease inter- and intrarater variability as described previously [73]. In the hippocampus and mesencephalon, all unique dilated VRS are counted. In 2013, we initiated the UNIVRSE (Uniform Neuro-Imaging of Virchow-Robin Spaces Enlargement)—consortium in order to investigate causes and consequences of VRS on a large scale [74].

## Main findings

### Cerebral small vessel disease

Already during the first round of MRI in the Rotterdam Study we learned that markers of ischemic small vessel disease such as WMLs and lacunar infarcts are highly prevalent in the elderly and that these relate to cardiovascular risk factors, such as hypertension or smoking [37, 75–84]. In the MRI scans obtained from 2005 onwards, we confirmed this frequent occurrence of WML and infarcts in the elderly and extended the prevalence and volume estimates to the middle aged population [68]. Moreover, we found these cardiovascular risk factors to be associated with a thinner cortex of the brain [85]. In addition to studying the role of above-mentioned cardiovascular risk factors in the development of ischemic small vessel disease, we also directly investigated associations of atherosclerosis with these markers. Using arterial calcification (measured with computed tomography) as an established marker of atherosclerosis, we found atherosclerotic calcification in various vessel beds, but especially in the intracranial vasculature, to be related to WMLs, lacunar infarcts, and brain atrophy [86, 87].

Besides focusing on its risk factors, we have also started investigating consequences of ischemic small vessel disease. In this light, we found that larger WML volumes and lacunar infarcts are associated with a higher risk of mortality [88], mild cognitive impairment [89], dementia [90], and a higher risk of stroke [91]. More recently, we found that larger WML volumes are associated with deterioration and incident impairment in daily functioning [92].

Besides the ischemic lesions in the context of cerebral small vessel disease, hemorrhagic lesions in the form of cerebral microbleeds have gained rapid interest [71]. To visualize cerebral microbleeds we use a dedicated 3D high-resolution T2* GRE sequence, which we found to improve the detection of cerebral microbleeds considerably in comparison with a conventional MRI sequence [93]. When we applied this high-resolution sequence in larger groups of participants, we found that cerebral microbleeds are present in 1 in 5 persons over age of 60 and in over 1 in 3 in persons aged 80 years and older [72, 94]. This prevalence is much higher than reported previously, which in part may be explained by the use of this dedicated MRI sequence. In a longitudinal study over an interval of 3–4 years, we found that 10 % of persons developed new microbleeds [95], and that this was highly dependent on the presence and amount of mircobleeds at baseline.

With regard to risk factors for microbleeds we consistently found that these vary according to the location of microbleeds in the brain. Cardiovascular risk factors and markers of ischemic small vessel disease are related to deep or infratentorial microbleeds, whilst *APOE* genotype relates to strictly lobar microbleeds [72, 94, 95]. This is indirect evidence that deep or infratentorial microbleeds result from arteriolosclerotic angiopathy, whereas strictly lobar microbleeds are caused by cerebral amyloid angiopathy [96].

The high prevalence of cerebral microbleeds and their potential link with bleeding-prone microangiopathy raised our interest in how these relate to antithrombotic drug use. We found that persons who had used or were using antithrombotic medication more often have cerebral microbleeds [97, 98]. Moreover, we found that the use of oral anticoagulants is associated with a higher incidence of microbleeds [99]. With regard to consequences of microbleeds, we found that the presence and amount of microbleeds increases the risk of stroke and mortality [100, 101]. Especially microbleeds in locations suggestive of amyloid angiopathy increase the risk of cerebral hemorrhages [101]. In addition, we found that microbleeds are associated with the progression of ischemic small vessel disease and loss of white matter structural integrity [102, 103]. Finally, we also showed that the presence of numerous microbleeds, especially in a strictly lobar location, is associated with worse cognitive performance. Adjustment for vascular risk factors and other imaging markers of small vessel disease did not alter this association [104], suggesting an independent role for microbleed-associated vasculopathy in cognitive impairment.

### Cerebral blood flow

Total cerebral blood flow (tCBF) and total brain perfusion (tCBF per 100 ml brain tissue) were measured with 2D phase contrast imaging as described above. We showed a close relationship between tCBF and markers of the microvasculature, e.g. retinal vessel diameters [75]. In 892 persons aged 60 years and older, we further showed that determinants of tCBF and total brain perfusion differ largely, due to the large influence of brain volume on tCBF values [105]. In a longitudinal study, we further investigated the relation between brain volume and tCBF, and our results indicate that brain atrophy likely causes the tCBF to decrease over time, rather than vice versa [106].

When investigating determinants of tCBF, we found that pulse pressure, body mass index, current smoking, and kidney function importantly contribute to variations in tCBF [105, 107]. Furthermore, persons with low total brain perfusion had significantly more WMLs compared to those with high total brain perfusion. The role of tCBF with cognitive performance appeared more complex with brain atrophy either confounding or mediating the association [108]. Finally, we found that the parenchymal CBF is higher is persons with migraine during the attack-free period when compared with persons without migraine, supporting the idea of sustained vascular differences in migraineurs [109].

### White matter microstructural integrity

We demonstrated that age-related changes in the normal-appearing white matter are primarily but not exclusively explained by white matter atrophy and formation of WMLs [110]. Using tract-specific analyses, we found specific white matter tracts including the commissural and limbic tracts, to be most prominently affected by aging. Furthermore, we found that white matter atrophy and WML formation related to loss of integrity in distinct brain regions, indicating that the two processes are not sequential events but are rather independent and thus pathophysiologically potentially different [53]. Finally, we found that white matter changes can already be quantified using DTI and FLAIR before actual WML develop. This suggests that WML develop gradually and that the WMLs that are visible only represent a small portion of the underlying white matter pathology [111].

We demonstrated that besides traditional cardiovascular risk factors [110], intracranial carotid artery atherosclerosis [87], cerebral microbleeds [102], and a reduced kidney function [112], are all associated with loss of white matter microstructural integrity.

We have found DTI parameters within WMLs and normal-appearing white matter to be associated with cognitive function, even when taking into account volume of WMLs and white matter atrophy [52]. This indicates that the deleterious effect of white matter changes on cognition not only depends on lesion burden or amount of atrophy, but also on characteristics that are not easily evaluated by conventional MRI.

### Imaging genetics

Since the advent of genome-wide association studies (GWAS) we have been involved in numerous studies in which the underlying genetics of various brain traits are investigated [113]. We have for example contributed to the identification of several single nucleotide polymorphisms (SNPs) associated with intracranial volume [114], and subcortical brain structures [115]. In collaboration with research partners in the CHARGE consortium [116], we performed a genome-wide association study of WML burden and were able to identify 6 risk-associated SNPs on chromosome 17q25 [117], which we subsequently replicated in a separate Rotterdam Scan Study cohort [41]. More recently, we identified another set of novel genetic loci implicating inflammation and glial proliferation in the development of WML [118]. Similar analyses have been undertaken for brain infarcts [119]. Moreover, we found that several risk variants of Alzheimer’s disease and frontotemporal dementia are also associated with smaller total brain volume and hippocampal volume [120], and the volume of temporal brain regions [121].

For additional EJE references on determinants of common neurological disorders see [122–136].

## Incidental findings

The large-scale acquisition of brain MRI comes with the detection of incidental findings [68]; abnormalities of potential clinical relevance unrelated to the purpose of the examination [137]. After scanning 2000 participants we found that the most frequent findings in the Rotterdam Scan Study are aneurysms (1.8 %) and benign primary tumors (meningiomas) (1.6 %) [68]. Currently, we are updating these numbers after scanning over 8500 participants, and evaluate the clinical management and natural course of these findings. This information may then serve as basis on which future recommendations for handling incidental findings in both the clinical setting and in research studies may be based.

For further EJE references on the handling of incidental findings in large-scale imaging studies see [138–141].

## Future perspectives

The Rotterdam Scan Study provides a unique environment to study the etiology of neurological diseases in the elderly. Over 5800 persons have already undergone brain MRI-scanning and in coming years repeated waves of follow-up examination will ensure a wealth of imaging data, especially from a longitudinal perspective. Until recently, the focus of our research has been mainly on the identification of risk factors and determinants of brain pathology. More recently, we have been examining the contribution of the various risk factors as discussed above and of novel risk factors with regard to the potential for prevention of stroke and dementia [142, 143]. In addition, we have also extended our scope of common neurological disorders to Parkinson’s disease (for further EJE references on Parkinson’s disease see [124, 144–149]), and for gait disorders see [92, 150–152]. In particular, our focus will include changes on brain imaging that relate to these conditions. Moreover, in the coming years, we plan to broaden our research in several ways, including the use of novel image sequences, novel post-processing techniques, and the identification of novel risk factors, and the mechanisms through which various lifestyle or genetic factors influence clinical outcomes through brain changes.

Currently, we are investigating the feasibility of adding perfusion imaging in the form of arterial spin labeling (ASL) to the scan protocol of the Rotterdam Scan Study. This will allow us to explore the interplay of structural and functional measures with (regional) brain perfusion. This is in particular of interest as perfusion may precede actual changes in structure or even function and could thus potentially be a very early marker of pathology.

Regarding new imaging markers, we are currently studying the prevalence and clinical correlates of small cortical brain infarcts in our population. In our initial report on these infarcts, we found that these are prevalent in 1.1 % of the population and are associated with cardiovascular risk factors [70]. Our interest in these small cortical infarcts arose from the current attention for cortical microinfarcts—microscopic small infarcts seen on pathologic exams—as potential new markers of cerebrovascular disease and indicators of cognitive impairment. Though larger in size, the small cortical infarcts that can be identified on MRI may be reflecting pathology similar to these microscopic lesions and are therefore of interest for further research.

Another important focus of our research will be on functional connectivity as new imaging marker in neurodegenerative disease. Currently, we are finalizing the rs-fMRI data analysis pipeline, and expect that end-2015 we will initiate the investigation of determinants and correlates of functional connectivity.

With regard to the MRI-scanner, we acknowledge that the ongoing hardware developments will necessitate upgrading of the scanner. Although there are no current plans to change the scanner, we foresee upgrading to a 3 T scanner in coming years.

Standardized and evaluated automated image processing techniques are crucial in exploiting the rich information that is available in population imaging data. They have enabled a transition from qualitative image interpretation into quantitative imaging. Quantitative imaging is non-trivial; it requires standardization and optimization in all the steps from data acquisition, to data analysis in structured reporting. In the next years, the number of validated quantitative image analysis techniques will further increase, also including more complex measures. Also, the results of these analyses will increasingly be stored in a standardized manner. This will enable a richer characterization of brain anatomy, pathology, connectivity and function. Relating these quantitative image measures to disease status, progression and events, will be a powerful tool for development of novel diagnostic and prognostic quantitative imaging biomarkers.

The standardization of image acquisition and processing protocols also implies that high quality reference data are being acquired. We are working on a novel IT-infrastructure in which we use the various standardized analysis pipelines to create a well-defined library of imaging biomarkers. We foresee two main advantages of this strategy. First, this standardized and well-defined library of biomarker analyses may be used by other researchers. Second, the high quality data provide unique reference databases on numerous biomarkers, which may eventually serve as basis for use in a clinical setting to contrast findings in an individual with a reference population.

As already mentioned before, the concomitant continuous monitoring of all participants in the Rotterdam Study ensures that we have a wealth of clinical data available, including cognitive performance [125] and the occurrence of dementia and stroke. In the coming years we intend to expand our research on how MRI markers of brain pathology relate to these clinical outcomes. Finally, we intend to expand our (inter)national collaborations in the field of population-imaging and imaging genetics to further unravel the causes and consequences of neurological diseases in the elderly.
